# The patterns and burden of multimorbidity in geriatric patients with prolonged use of addictive medications

**DOI:** 10.1007/s40520-021-01791-5

**Published:** 2021-02-18

**Authors:** Socheat Cheng, Tahreem Ghazal Siddiqui, Michael Gossop, Torgeir Bruun Wyller, Espen Saxhaug Kristoffersen, Christofer Lundqvist

**Affiliations:** 1 Division of Health Services Research and Psychiatry (AHUSKHP), Faculty of Medicine, Institute of Clinical Medicine, University of Oslo, PO Box 1000, 1478 Lørenskog, Norway; 2 Health Services Research Unit (HØKH), Akershus University Hospital, Lørenskog, Norway; 3 Faculty of Medicine, Institute of Clinical Medicine, University of Oslo, Campus Ahus, Lørenskog, Norway; 4 National Addiction Centre, Institute of Psychiatry, Psychology and Neuroscience, King’s College London, London, UK; 5 Department of Geriatric Medicine, Faculty of Medicine, Institute of Clinical Medicine, University of Oslo, Oslo, Norway; 6 Department of General Practice, Institute of Health and Society, University of Oslo, Oslo, Norway; 7 Department of Neurology, Akershus University Hospital, Lørenskog, Norway

**Keywords:** Older patients, Prescription drug overuse, Chronic diseases, Medication safety

## Abstract

**Background:**

Multimorbidity and prolonged use of addictive medications are prevalent among older patients, and known to increase the risk of adverse drug events. Yet, the relationship between these two entities has remained understudied.

**Aims:**

This study explored the association between multimorbidity burden and prolonged use of addictive medications in geriatric patients, adjusted for clinically important covariates. Furthermore, we identified comorbidity patterns in prolonged users.

**Methods:**

We conducted a cross-sectional study on a consecutive sample of 246 patients, aged 65–90 years, admitted to a large public university hospital in Norway. We defined prolonged use of addictive medications as using benzodiazepines, opioids and/or z-hypnotics beyond the duration recommended by clinical guidelines (≥ 4 weeks). Multimorbidity was assessed with the Cumulative Illness Rating Scale for Geriatrics (CIRS-G), based on diagnoses made by independent physicians.

**Results:**

Compared to non-prolonged use, prolonged use was significantly more common among patients who had psychiatric (19/27, 70%), liver (19/22, 86%), upper gastrointestinal tract (21/32, 66%), musculoskeletal (52/96, 54%), or nervous system disorders (46/92, 50%). Patients with prolonged use had a higher multimorbidity burden than those without such use (CIRS-G score, mean = 7.7, SD = 2.7 versus mean = 4.6, SD = 2.2, *p* < 0.001). Multivariable logistic regression indicated a significant association between multimorbidity burden and prolonged addictive medication use (OR = 1.72, 95% CI 1.42–2.08). Predictive margins postestimation showed a systematic increase in the predicted CIRS-G scores when the number of addictive drug used increases.

**Conclusions:**

Multimorbidity is strongly associated with prolonged use of addictive medications. Multiple substance use may aggravate disease burden of older patients.

**Supplementary Information:**

The online version contains supplementary material available at 10.1007/s40520-021-01791-5.

## Introduction

Medications with abuse liability such as benzodiazepines, opioid analgesics, and Z-hypnotics are commonly prescribed for the management of anxiety, pain and insomnia [1, 2]. However, due to high-addictive potentials and risk of other serious complications (e.g. falls, fractures and cognitive impairment), these drugs are listed in various medication appropriateness criteria (e.g. NORGEP, Beers and STOPP criteria) as potentially inappropriate for older people [3]. Clinical guidelines only recommend short-term use (< 4 weeks) [4, 5]. Nonetheless, prolonged use is widespread [6, 7]. In Norway, the prevalence has remained high since the past decade. Recent studies revealed high proportions of geriatric patients on persistent and concomitant use of addictive medications [8–10].

Multimorbidity, defined as co-occurrence of two or more diseases in one person, is also prevalent among older adults [11, 12]. Not only is it a predictor of poorer health-related quality of life, multimorbidity also increases healthcare utilization, and can be difficult to manage [13]. The presence of multiple conditions challenges clinicians to provide tailored care and anticipate problems caused by using diverse medications or treatment forms [14].

Although multimorbidity and prolonged use of addictive medications are common in ageing populations, the intersection between these two entities is poorly understood. Very few studies have examined this topic specifically among older patients. Also, those conducted generally aimed to assess the likelihood of being prescribed potentially addictive medications rather than the effect of multimorbidity on prolonged drug use [15, 16]. Moreover, insights regarding diseases that commonly coexist with prolonged use are scarce. Addressing these knowledge gaps is important to guide future interventions for optimizing health outcomes of older people with multiple conditions.

We aimed therefore to explore the relationship between multimorbidity burden and prolonged use of addictive medications in geriatric patients and to identify comorbidity patterns of patients on extended use of such drugs.

## Materials and methods

### Study design, setting and recruitment

This study was cross-sectional. Participants were recruited consecutively between May 2017 and September 2018 from the departments of geriatrics, general medicine and neurology of a large public university hospital in Norway (Akershus universitetssykehus). The study was open to all patients aged between 65 and 90 years, regardless of sociodemographic background. We did not include patients who were critically ill or in palliative treatment (defined by clinicians at the medical wards). Exclusion criteria included incapacity to give informed consent (indicated by a Mini-Mental State Examination (MMSE) score ≤ 21) [17]; established diagnosis of severe depression, dementia or psychotic disorders; severe visual or hearing impairment; and insufficient knowledge of the Norwegian language. These criteria were predefined and registered prior to the initiation of the study (NCT03162081), and generally based on ethical considerations and a wish not to burden very ill patients further and to minimize reporting bias. No information on disease profiles or medication use was made available to our research team prior to and during the recruitment process as this was stored in the electronic medical records (EMRs), which could only be accessed after obtaining written informed consent from the recruited participants.

### Study variables and assessment procedure

#### Main variables

The main variables in this study were prolonged use of addictive medications and multimorbidity. We defined prolonged use as using benzodiazepines, opioid analgesics and/or Z-hypnotics beyond the duration recommended by clinical guidelines (≥ 4 consecutive weeks) [4, 5]. Data for this variable were collected through reviewing EMRs and crosschecked with the patients and referral documents. We assessed patients’ multimorbidity using the Cumulative Illness Rating Scale for Geriatrics (CIRS-G), based on diagnoses made by physicians at the medical wards, who were not in the research team. The scale contains 14 organ-specific categories, showing comorbidity patterns of a patient. Each category can be rated from 0 to 4 depending on the severity of the conditions diagnosed. Adding up the scores for all these categories gives a total CIRS-G score, ranging from 0 to 56. Higher total CIRS-G scores indicate higher multimorbidity burden [18].

#### Adjustment variables

Based on our previous findings [10], socioeconomic variables included in the analyses were sex, age (in years), educational attainment (basic, secondary, and higher education), annual income (< 200,000, 200,000–349,000, and ≥ 350,000 Norwegian krone per year) and living situation (living alone versus living with others). Clinical variables comprised pain intensity, anxiety and depression symptoms. Pain intensity was measured on a 10 cm Visual Analogue Scale (VAS), indicating how much pain the respondent is currently feeling. A higher score indicates more intense pain [19]. Anxiety and depression symptoms were assessed with the Hospital Anxiety and Depression Scale (HADS). HADS has two subscales: anxiety (HADS-A) and depression (HADS-D). Each subscale has 7 items and each item is scored from 0 to 3. Thus, the total score for each subscale varies between 0 and 21 [20]. The optimal cutoff value for older patients remains to be established. As suggested by Bell et al. (2016), we used the total HADS-A/D scores as continuous variables to avoid misclassification bias. Higher scores indicate higher levels of anxiety and depressive symptoms [21]. Data for these variables were collected through questionnaires completed by the patients.

### Statistical analysis

Summary statistics (mean, standard deviation (SD); median, interquartile range (IQR); and frequency) were used to describe characteristics of participants. We assessed differences between groups using *t* test, chi-squared test, and Kruskal–Wallis test as appropriate. Bivariable and multivariable logistic regression analyses were performed to identify associations between the dependent and the explanatory variables. Prolonged use of addictive medications was chosen as the outcome variable and multimorbidity burden as the main explanatory variable. The strength of the associations is presented as odds ratios (OR) and 95% confidence intervals (CI). We also performed bootstrapped sensitivity analysis using 500 replications to confirm the main result. Furthermore, we used predictive margins postestimation (after fitting multiple linear regression) to check dose–response patterns between the number of addictive drugs and disease burden. STATA/SE16.0 software was used for all statistical analyses.

## Results

### Participants

**Fig. 1:**
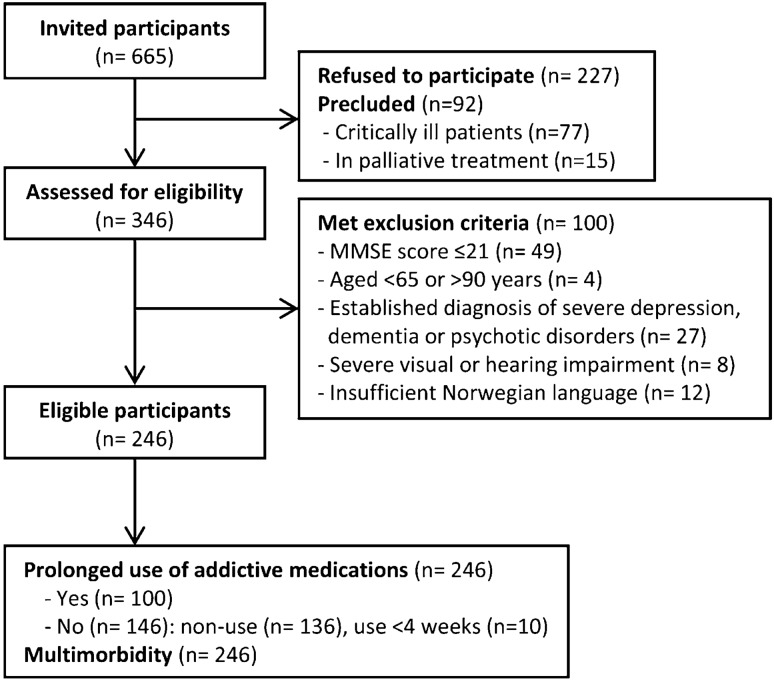
Flow of participants through the study

In total, we approached 665 patients at the medical wards. Of these, 346 patients consented to participate whereas 227 refused the invitation and 92 were precluded due to being critically ill or in palliative treatment. Of the 346 patients who consented to participate, 100 were excluded based on our predefined criteria: aged < 65 or > 90 years (*n* = 4), MMSE score ≤ 21 (*n* = 49), established diagnosis of severe depression, dementia or psychotic disorders (*n* = 27), severe visual or hearing impairment (*n* = 8), and insufficient knowledge of the Norwegian language (*n* = 12). We therefore had 246 eligible participants. The flow of participants through the study is shown in Fig. 1.

Comparing characteristics of participants (*n* = 246) versus non-participants (*n* = 369) showed sex differences. The non-participant group comprised more males (205/369, 56%) than the participant group (109/246, 44%, *p* = 0.003). The two groups did not differ significantly in age (mean = 76.6, SD = 7.4 versus mean = 76.6, SD = 6.6, *p* = 0.09). Participation rates across the three departments were comparable (38.8% geriatrics, 39% general medicine, and 41% neurology, *p* = 0.82). As defined by the responsible ethical committees in Norway, we had no possibility of obtaining multimorbidity and other clinical data from patients who did not consent to participation in the study.

We had complete data for the two main variables (prolonged medication use and CIRS-G multimorbidity), medication groups, age, sex and living situations. The percentages of missing data for the remaining variables were: 6% for education (14/246), 7% for anxiety, depression and pain (17/246), and 16% for income (39/246).

Table 1 shows characteristics of the study sample. Of the 246 eligible participants, 100 were identified as being on prolonged use of addictive medications (≥ 4 consecutive weeks). This included 70 patients who exclusively used one of the three focussed medication groups (21 opioid users, 7 benzodiazepine users, and 42 Z-hypnotic users); while 30 concurrently used several types of these medications. The median duration of use for opioids analgesics was 42 weeks (IQR = 11–113); while that of for benzodiazepines and Z-hypnotics were 51 weeks (IQR = 17–78) and 52 weeks (IQR = 15–77), respectively.

**Table 1:** Characteristics of the study sample

Items, number (%) unless stated otherwise	Prolonged use of addictive medication		
	No (*n* = 146)	Yes (*n* = 100)	*P* value
Sex			
Female	71 (52)	66 (48)	**0.01**
Male	75 (69)	34 (31)	
Age in years, mean (SD)	75 (6.4)	78 (6.5)	** < 0.001**
Educational attainment			
Basic education	16 (35)	30 (65)	**0.001**
Secondary education	64 (67)	31 (33)	
Higher education	58 (64)	33 (36)	
Annual income (Norwegian krone)			
< 200,000	8 (38)	13 (62)	**0.001**
200 000–349 000	42 (49)	43 (51)	
≥ 350,000	72 (71)	29 (29)	
Living situations			
Living with others	87 (66)	45 (34)	**0.03**
Living alone	59 (52)	55 (48)	
Hospital anxiety and depression scale (HADS)			
Anxiety score (HADS-A), median (IQR)	4 (1–6)	4 (2–8)	0.17
Depression score (HADS-D), median (IQR)	3 (1–6)	4 (2–7)	** < 0.001**
Pain intensity (VAS in centimetres), median (IQR)	0.7 (0.03–2.7)	2.9 (0.5–6.1)	** < 0.001**

Bold values indicate *P* value < 0.05

*VAS* visual analogue scale, *SD* standard deviation, *IQR* interquartile range

### Multimorbidity patterns

**Fig. 2:**
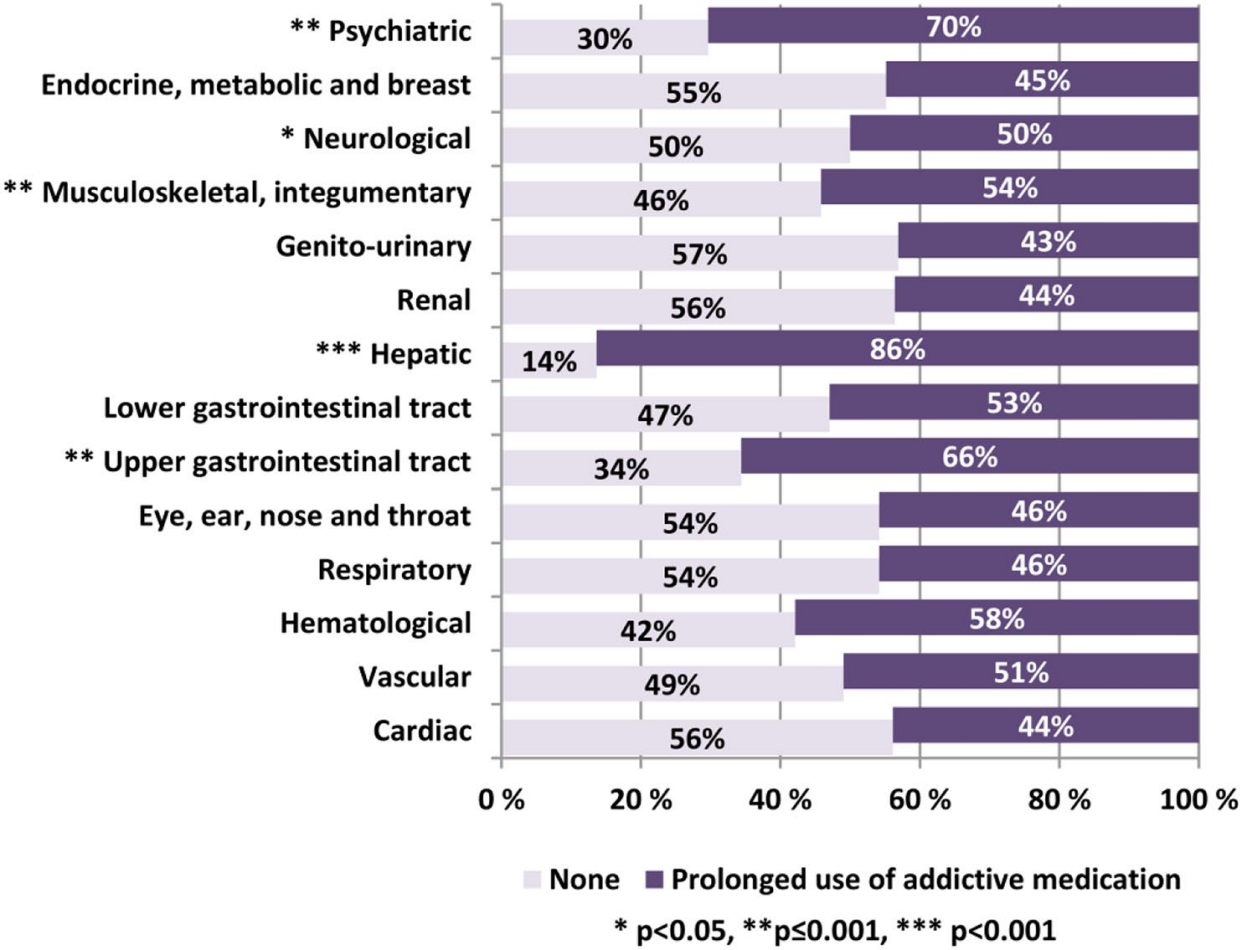
Multimorbidity patterns

Figure 2 depicts multimorbidity patterns of patients with versus without prolonged use of addictive medications. Prolonged use of addictive medications was significantly more common among patients diagnosed with psychiatric disorders (e.g. anxiety, depression, delirium and personality disorders); and/or morbidity in liver (e.g. hepatitis, cirrhosis, cholecystitis and carcinoma), upper gastrointestinal tract (e.g. ulcer, dysphagia, bleeding and cancer), musculoskeletal (e.g. arthritis, skin infection, melanoma and osteoporosis), or nervous systems (e.g. chronic headache, vertigo, Parkinson's disease and multiple sclerosis). Post hoc analyses adjusted for relevant covariates showed significant associations between prolonged drug use and the presence of the following disease categories: upper gastrointestinal tract (OR = 2.78, 95% CI 1.05–7.37), liver (OR = 24.62, 95% CI 4.46–135.94), musculoskeletal system (OR = 2.05, 95% CI 1.01–4.22) and nervous system (OR = 2.71, % CI 1.27–5.78). More details are shown in Online Appendices 1–4.

### Multimorbidity burden

The mean score of multimorbidity burden for the study sample was 5.89 (SD = 2.84).

Patients with prolonged use of addictive medications had higher multimorbidity burden than those without such use (CIRS-G score mean = 7.70, SD = 2.74 versus Mean = 4.64, SD = 2.15, *p* < 0.001). The burden was comparable for those who used only one type versus several types of addictive medications (CIRS-G mean = 7.88, SD = 2.78 versus Mean = 7.27, SD = 2.65, *p* = 0.30). Kruskal–Wallis H test indicated no significant differences in multimorbidity burden between users of the three groups of medications, *χ*^2^(2) = 0.65, *p* = 0.72, with a mean rank CIRS-G scores of 34 for opioid users, 31 for benzodiazepine users and 37 for Z-hypnotic users.

**Fig. 3:**
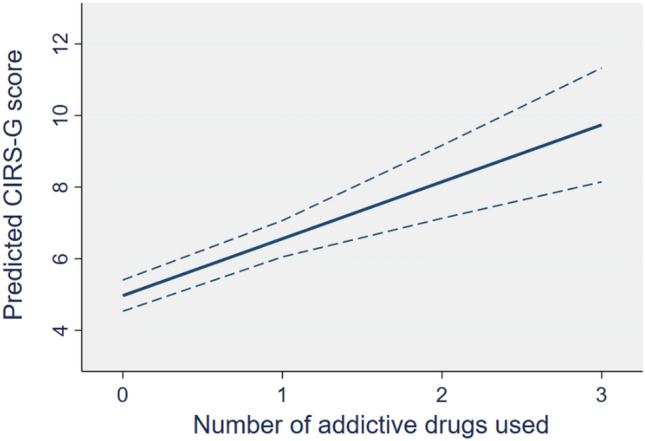
Dose–response patterns between the number of addictive drugs and disease burden

Table 2 presents bivariable and multivariable logistic regression models of the associations between the two main variables and other covariates. Multimorbidity burden was significantly associated with prolonged use of addictive medications (OR = 1.72, 95% CI 1.42–2.08), adjusted for sex, age, educational attainment, annual income, living situations, pain, anxiety and depression scores. In addition, the model showed a significant association between pain intensity and prolonged use. Also, compared to those with the highest income, patients who earned below 200 000 NOK/year and 200,000–349,000 NOK/year had higher odds for prolonged use of addictive medications. Bootstrapped sensitivity analysis yielded consistent results (Online Appendix 5). Subsequent multiple linear regression analysis showed a significant relationship between the number of addictive drugs used and CIRS-G scores (disease burden), *β* = 1.58 (95% CI 1.00–2.19). Postestimation using predictive margins, as shown in Fig. 3, indicated a systematic increase in disease burden when the number of substances used increases. Predicted values of disease burden when the number of drugs used were 0, 1, 2 and 3 were 4.97 (95% CI 4.53–5.41), 6.56 (95% CI 6.05–7.07), 8.15 (95% CI 7.13–9.17), and 9.74 (95% CI 8.15–11.33), respectively.

**Table 2:** The relationship between multimorbidity burden and prolonged use of addictive medications

Covariates	Bivariable models		Multivariable model	
	OR (95% CI)	*P* value	Adjusted OR (95% CI)	*P* value
Multimorbidity burden	1.69 (1.46–1.96)	** < 0.001**	1.72 (1.42–2.08)	** < 0.001**
Age	1.08 (1.04–1.12)	** < 0.001**	1.06 (0.99–1.14)	0.09
Sex				
Male (reference)				
Female	2.05 (1.21–3.47)	**0.01**	1.89 (0.80–4.46)	0.15
Educational attainment				
Basic education (reference)				
Secondary education	0.26 (0.12–0.54)	** < 0.001**	0.36 (0.11–1.15)	0.08
Higher education	0.30 (0.14–0.64)	**0.002**	0.59 (0.18–1.89)	0.37
Annual income (Norwegian krone)				
≥ 350 000 (reference)				
200,000–349,999	2.54 (1.39–4.66)	**0.003**	2.60 (1.06–6.37)	**0.04**
< 200 000	4.03 (1.51–10.75)	**0.01**	11.21 (2.33–53.96)	**0.003**
Living situations				
Living with others (reference)				
Living alone	1.80 (1.08–3.01)	**0.03**	0.64 (0.25–1.60)	0.34
Anxiety score (HADS-A)	1.07 (0.99–1.15)	0.08	1.05 (0.92–1.21)	0.44
Depression score (HADS-D)	1.16 (1.06–1.26)	**0.001**	1.03 (0.89–1.19)	0.68
Pain intensity (VAS), per cm	1.25 (1.13–1.38)	** < 0.001**	1.31 (1.13–1.52)	** < 0.001**

Bold values indicate *P* value < 0.05

*VAS* visual analogue scale, *HADS* hospital anxiety and depression scale

## Discussion

We found that patients on prolonged use of addictive medications had higher multimorbidity burden, compared to those without such use. Most of them had co-diagnosis of psychiatric disorders and/or diseases in liver, upper gastrointestinal tract, musculoskeletal and integumentary, or nervous systems. Higher multimorbidity burden was strongly associated with higher odds of being on prolonged use of addictive medications, even after controlling for important confounders and confirming by sensitivity analysis.

To our knowledge, our study is the first to comprehensively assess comorbid conditions associated with prolonged use of prescribed addictive medications in geriatric patients across the organ systems. We found that such prolonged use coexisted with various specific types of illnesses. This suggests that the effort to detect problematic use of addictive medications among older patients should not be limited to those with psychiatric disorders, but widened to include also other groups of somatic comorbidities as shown. The significant relationships between lower income, intense pain and prolonged use, found in our study are in accordance with previous studies [22, 23]. Socioeconomically disadvantaged people might have been exposed to stressful events and sleep difficulties, leading to the use of benzodiazepines and/or z-drugs [22]. It has been shown that persistent use of opioids can cause increased pain intensity (hyperalgesia) though actual mechanisms remain unknown [23].

Research in Iceland showed that the majority of those who received benzodiazepine and/or Z-hypnotic prescriptions were multimorbid; and that those with multimorbidity were 15 times more likely than those without to be prescribed these drugs [24]. Another recent study focussing on older hospitalized patients in San Francisco, also reported that patients with higher multimorbidity burden tended to receive prescriptions of opioid analgesics at discharge, adjusted for sociodemographic variables, and pain [15]. Our results corroborate these findings, and also suggest that multimorbidity is not only associated with prescribing but also with long-term use of addictive medications. It has been proposed that simultaneously having many diseases may evoke feelings of worry, discomfort and sleep disturbances, which may lead to the start of opioid analgesics, benzodiazepines or Z-hypnotic prescriptions [15, 24]. Hypothetically, the progression towards persistent use in geriatric patients with multimorbidity may be reinforced by many factors, including inappropriate prescribing practices, users’ attitudes, poor doctor-patients communications, drug dependence and psychiatric side effects. It is also possible that side effects of prolonged use of the drugs may intensify the burden of multimorbidity. To guide clinical practice, future research, especially longitudinal studies, is encouraged to clarify potential causative pathways and dose–response relationship.

Older patients with multimorbidity may have complex medication regimens, which can lead to undesirable events [25]. Also, excessive use of opioid analgesics and sedative-hypnotic drugs can cause harm. Feng et al. (2017) explained that therapeutic effects and toxicity of opioids can be altered when co-medicated with other drugs; and that those metabolized by the cytochrome P450 (CYPs) system tend to be associated with drug–drug interactions [26]. Taking benzodiazepines, opioids and/or other central nervous system depressant agents simultaneously can also cause oversedation and respiratory failure [27]. Because of age-related changes in pharmacodynamics and pharmacokinetics, prolonged use of these drugs may be even more problematic for older patients with multimorbidity. Pertaining to this issue, the National Institute for Health and Care Excellence (NICE) guidance recommends establishing patients’ current health status and reviewing medicines as critical steps to offer suitable care [28]. Furthermore, the Centers for Disease Control and Prevention (CDC) guideline for prescribing opioids for chronic pain recommends an assessment of benefits versus harms of the drugs within the first few weeks [29]. Based on these recommendations and our findings, it seems advisable that physicians reassess disease burden and screen for overuse of addictive drugs among older patients at the latest four weeks after initiation.

The strength of this study is that it focussed specially on older patients when assessing the relationship between multimorbidity and prolonged use of addictive medications. Nonetheless, it has some limitations. Using a consecutive hospital-based sample limits the generalizability of the study findings. However, while random sampling was less practical in our situation, we tried to form a sample which was as representative as possible through consecutive sampling. Moreover, our sample constitutes patients admitted to medical wards, regardless of their socioeconomic background and for a variety of health problems, and as such should be reasonably representative for hospital populations of older patients in Scandinavia. Another limitation is the use of cross-sectional design, which makes it difficult to determine the direction of causality. Thus, the associations observed can be bidirectional. In addition, there were many patients who refused to participate or who met the exclusion criteria. It may be possible that patients with problematic use of addictive medications were over-represented among those who were not willing to participate. Hence, we cannot exclude the possibility that our sample was biased towards milder cases among those admitted to hospital. This may also suggest that if the severely ill patients had been included, the effect size could have been greater.

## Conclusions

Having higher multimorbidity burden increases the odds of being on prolonged use of addictive medications, or vice versa. Multiple substance use may aggravate disease burden of older patients. While opioid analgesics, benzodiazepines and Z-hypnotics offer rapid relief, evidence for the risks of serious complications associated with long-term use is mounting. Thus, to reduce susceptibility to adverse drug events for older patients, physicians should remain vigilant, and whenever suitable, as part of the treatment, also consider removal of drugs with possible adverse effects.

## Availability of data and material

Data are not publicly available due threats to subject privacy, but are available from the Data Protection Officer at Akershus University Hospital and Division of Health Services Research and psychiatry of the University of Oslo for researchers who meet the criteria for access to confidential data. They may also contact the corresponding author.

## Supplementary Information

Below is the link to the electronic supplementary material.

Supplementary file1 (PDF 534 KB)
