# Supplementary material for: The patterns and burden of multimorbidity in geriatric patients with prolonged use of addictive medications
Source: Aging Clin Exp Res. 2021 Feb 18;33(10):2857–64. doi: 10.1007/s40520-021-01791-5 (PMC8531043; doi:10.1007/s40520-021-01791-5)
Supplement: Supplementary file 1 — Supplementary file1 (PDF 534 KB) [file 40520_2021_1791_MOESM1_ESM.pdf]

Appendix 1 Association between diseases in upper gastrointestinal tract and prolonged use of addictive medications

Covariates	Logistic regression models			
	**Bivariable models**		**Multivariable model**	
	**OR (95% CI)**	**P-value**	**Adjusted OR (95% CI)**	**P-value**
**Diseases in UGIT**				
Absence (reference)				
Presence	3.26 (1.49 to 7.12)	**0.003**	2.78 (1.05 to 7.37)	**0.04**
**Age**	1.08 (1.04 to 1.12)	**<0.001**	1.06 (1.01 to 1.13)	**0.03**
**Sex**				
Male (reference)				
Female	2.05 (1.21 to 3.47)	**0.01**	1.79 (0.85 to 3.74)	0.12
**Educational attainment**				
Basic education (reference)				
Secondary education	0.26 (0.12 to 0.54)	**<0.001**	0.29 (0.11 to 0.80)	**0.02**
Higher education	0.30 (0.14 to 0.64)	**0.002**	0.38 (0.14 to 1.02)	0.06
**Annual income** (Norwegian krone)				
< 200 000 (reference)				
200 000–349 999	0.63 (0.24 to 1.68)	0.36	0.35 (0.10 to 1.19)	0.09
≥ 350 000	0.25 (0.093 to 0.66)	**0.01**	0.19 (0.05 to 0.66)	**0.01**
**Living situations**				
Living with others (reference)				
Living alone	1.80 (1.08 to 3.01)	**0.03**	0.58 (0.26 to 1.32)	0.20
**Anxiety score** (HADS-A)	1.07 (0.99 to 1.15)	0.08	1.01 (0.90 to 1.14)	0.81
**Depression score** (HADS-D)	1.16 (1.06 to 1.26)	**0.001**	1.12 (0.99 to 1.27)	0.07
**Pain intensity** (VAS), per cm	1.25 (1.13 to 1.38)	**<0.001**	1.31 (1.15 to 1.49)	**<0.001**

Abbreviations: VAS – visual analogue scale; HADS – hospital anxiety and depression scale
UGIT– upper gastrointestinal tract

Appendix 2 Association between diseases in the liver and prolonged use of addictive medications

Covariates	Logistic regression models			
	Bivariable models		Multivariable model	
	OR (95% CI)	P-value	Adjusted OR (95% CI)	P-value
**Diseases in the liver**				
Absence (reference)				
Presence	11.18 (3.21 to 38.94)	**<0.001**	24.62 (4.46 to 135.94)	**<0.001**
**Age**	1.08 (1.04 to 1.12)	**<0.001**	1.09 (1.02 to 1.16)	**0.01**
**Sex**				
Male (reference)				
Female	2.05 (1.21 to 3.47)	**0.01**	2.08 (0.94 to 4.56)	0.07
**Educational attainment**				
Basic education (reference)				
Secondary education	0.26 (0.12 to 0.54)	**<0.001**	0.26 (0.09 to 0.75)	**0.01**
Higher education	0.30 (0.14 to 0.64)	**0.002**	0.33 (0.12 to 0.93)	**0.04**
**Annual income** (Norwegian krone)				
< 200 000 (reference)				
200 000–349 999	0.63 (0.24 to 1.68)	0.36	0.47 (0.12 to 1.81)	0.27
≥ 350 000	0.25 (0.093 to 0.66)	**0.01**	0.22 (0.06to 0.92)	**0.04**
**Living situations**				
Living with others (reference)				
Living alone	1.80 (1.08 to 3.01)	**0.03**	0.62 (0.27 to 1.46)	0.28
**Anxiety score** (HADS-A)	1.07 (0.99 to 1.15)	0.08	1.04 (0.92 to 1.18)	0.51
**Depression score** (HADS-D)	1.16 (1.06 to 1.26)	**0.001**	1.13 (0.99 to 1.28)	0.06
**Pain intensity** (VAS), per cm	1.25 (1.13 to 1.38)	**<0.001**	1.28 (1.12 to 1.47)	**<0.001**

Abbreviations:
VAS – visual analogue scale; HADS – hospital anxiety and depression scale

Appendix 3 Association between diseases in musculoskeletal system prolonged use of addictive medications

Covariates	Logistic regression models			
	Bivariable models		Multivariable model	
	OR (95% CI)	P-value	Adjusted OR (95% CI)	P-value
**Diseases in musculoskeletal system**				
Absence (reference)				
Presence	2.51 (1.48 to 4.25)	**0.001**	2.05 (1.01 to 4.22)	**0.04**
**Age**	1.08 (1.04 to 1.12)	**<0.001**	1.07 (1.01 to 1.14)	**0.02**
**Sex**				
Male (reference)				
Female	2.05 (1.21 to 3.47)	**0.01**	1.7 (0.81 to 3.55)	0.16
**Educational attainment**				
Basic education (reference)				
Secondary education	0.26 (0.12 to 0.54)	**<0.001**	0.28 (0.10 to 0.76)	**0.01**
Higher education	0.30 (0.14 to 0.64)	**0.002**	0.34 (0.12 to 0.92)	**0.03**
**Annual income** (Norwegian krone)				
< 200 000 (reference)				
200 000–349 999	0.63 (0.24 to 1.68)	0.36	0.41 (0.12 to1.44)	**0**.17
≥ 350 000	0.25 (0.093 to 0.66)	**0.01**	0.20 (0.05 to 0.72)	**0.01**
**Living situations**				
Living with others (reference)				
Living alone	1.80 (1.08 to 3.01)	**0.03**	0.55 (0.24 to 1.23)	0.14
**Anxiety score** (HADS-A)	1.07 (0.99 to 1.15)	0.08	1.02 (0.90 to 1.15)	0.76
**Depression score** (HADS-D)	1.16 (1.06 to 1.26)	**0.001**	1.12 (0.99 to 1.27)	0.06
**Pain intensity** (VAS), per cm	1.25 (1.13 to 1.38)	**<0.001**	1.26 (1.11 to 1.43)	**<0.001**

Abbreviations:
VAS – visual analogue scale; HADS – hospital anxiety and depression scale

Appendix 4 Prolonged use of addictive medications and the presence of diseases in nervous system

Covariates	Logistic regression models			
	Bivariable models		Multivariable model	
	OR (95% CI)	P-value	Adjusted OR (95% CI)	P-value
**Diseases in nervous system**				
Absence (reference)				
Presence	1.85 (1.09 to 3.13)	**0.02**	2.71 (1.27 to 5.78)	**0.01**
**Age**	1.08 (1.04 to 1.12)	**<0.001**	1.08 (1.02 to 1.15)	**0.01**
**Sex**				
Male (reference)				
Female	2.05 (1.21 to 3.47)	**0.01**	1.92 (0.91 to 4.04)	0.09
**Educational attainment**				
Basic education (reference)				
Secondary education	0.26 (0.12 to 0.54)	**<0.001**	0.31 (0.11 to 0.85)	**0.02**
Higher education	0.30 (0.14 to 0.64)	**0.002**	0.32 (0.12 to 0.88)	**0.03**
**Annual income** (Norwegian krone)				
< 200 000 (reference)				
200 000–349 999	0.63(0.24 to 1.68)	0.36	0.38 (0.11 to 1.31)	0.13
≥ 350 000	0.25(0.093 to 0.66)	**0.01**	0.20 (0.05 to 0.72)	**0.01**
**Living situations**				
Living with others (reference)				
Living alone	1.80 (1.08 to 3.01)	**0.03**	0.55 (0.25 to 1.25)	0.16
**Anxiety score** (HADS-A)	1.07 (0.99 to 1.15)	0.08	0.99 (0.88 to1.13)	0.98
**Depression score** (HADS-D)	1.16 (1.06 to 1.26)	**0.001**	1.15 (1.01 to 1.30)	**0.04**
**Pain intensity** (VAS), per cm	1.25 (1.13 to 1.38)	**<0.001**	1.33 (1.16 to 1.52)	**<0.001**

Abbreviations:
VAS – visual analogue scale; HADS – hospital anxiety and depression scale

Appendix 5 Bootstrapping sensitivity analysis (500 replications)

Covariates	Bivariable model		Multivariable model	
	OR (95% CI)	P-value	Adjusted OR (95% CI)	P-value
**Multimorbidity burden**	1.69 (1.47 to 1.94)	**<0.001**	1.72 (1.48 to 1.99)	**<0.001**
**Age**	1.08(1.04 to 1.12)	**<0.001**	1.06 (0.97 to 1.16)	0.20
**Sex**				
Male (reference)				
Female	2.05 (1.20 to 3.51)	**0.01**	1.89 (0.68 to 5.21)	0.22
**Educational attainment**				
Basic education (reference)				
Secondary education	0.26 (0.12 to 0.57)	**0.001**	0.36 (0.06 to 2.34)	0.28
Higher education	0.30 (0.14 to 0.67)	**0.003**	0.59 (0.09 to 3.75)	0.57
**Annual income** (Norwegian krone)				
≥ 350 000 (reference)				
200 000–349 999	2.54 (1.39 to 4.67)	**0.003**	2.60 (1.07 to 6.30)	**0.04**
< 200 000	4.03 (1.36 to 11.98)	**0.01**	11.21 (2.91 to 43.23)	**<0.001**
**Living situation**				
Living with others (reference)				
Living alone	1.80 (1.06 to 3.06)	**0.03**	0.64 (0.24 to 1.71)	0.37
**Anxiety score** (HADS-A)	1.07 (0.99 to 1.16)	0.11	1.05 (0.89 to 1.25)	0.54
**Depression score** (HADS-D)	1.16 (1.07 to 1.25)	**<0.001**	1.03 (0.83 to 1.28)	0.79
**Pain intensity** (VAS), per cm	1.25 (1.13 to 1.38)	**<0.001**	1.31 (1.08 to 1.59)	**0.01**

Abbreviations:
VAS – visual analogue scale; HADS – hospital anxiety and depression scale
